# Norcantharidin Suppresses Colon Cancer Cell Epithelial-Mesenchymal Transition by Inhibiting the αvβ6-ERK-Ets1 Signaling Pathway

**DOI:** 10.1038/srep20500

**Published:** 2016-02-05

**Authors:** Cheng Peng, Zequn Li, Zhengchuan Niu, Wei Niu, Zongquan Xu, Huijie Gao, Weibo Niu, JiaYong Wang, Zhaobin He, Chao Gao, Pengfei Lin, Michael Agrez, Zongli Zhang, Jun Niu

**Affiliations:** 1 Department of General Surgery, Qilu Hospital, Shandong University, Jinan, Shandong, China; 2 Department of Hepatic Oncology, Jiangxi Provincial Cancer Hospital, Nanchang, Jiangxi, China; 3 Newcastle Bowel Cancer Research Collaborative, The University of Newcastle, Callaghan, New South Wales, Australia

## Abstract

Norcantharidin (NCTD) is an efficacious anti-cancer drug that has been used in China for many years, but its underlying mechanism of action is still not fully understood. In the present study, we found that NCTD could induce morphological changes in colon cancer cells, causing a transition from a spindle-shaped morphology to a typical round or oval shape, which was indicative of a mesenchymal-epithelial transition (MET) process. Next, we investigated the mechanism by which NCTD induced the MET process. Using a transwell assay, we found that NCTD could suppress the migratory and invasive ability of colon cancer cells in a dose-dependent manner. Moreover, NCTD suppressed the expression of integrin αvβ6, MMP-3, and MMP-9 as well as the polymerization of F-actin, further supporting its suppressive effect on migratory and invasive ability. Furthermore, the expression of αvβ6, N-cadherin, vimentin and phosphorylated ERK was decreased, while the expression of E-cadherin was up-regulated. We verified that phosphorylated Ets1 was down-regulated substantially after treatment with NCTD. Taken together, our data demonstrated that NCTD could inhibit the EMT process of colon cancer cells by inhibiting the αvβ6-ERK-Ets1 signaling pathway. This study revealed part of the mechanism through which NCTD could reverse the EMT process in colon cancer.

Cantharidin is an efficacious anti-tumor drug extracted from blister beetles (*Mylabris phalerata* Pall) that has been used in China for over 2,000 years. The clinical application of cantharidin has been restricted due to its significant side effects, such as urinary system toxicity^1^. Recently, congener drugs with fewer adverse reactions, such as N-hydroxycantharidimide and sodium cantharidinate, have been synthesized. A new type of congener drug, norcantharidin (NCTD), is a demethylated form of cantharidin that is synthesized using furane and maleic anhydride through additive reactions. The urinary system toxicity of this drug is significantly reduced due to the removal of two methyl groups, while its anti-cancer activity remain unaffected. Researchers have shown that NCTD could inhibit the proliferation of several tumor cell lines *in vivo* and *in vitro* via different mechanisms^2^,^3^.

Our previous study showed that NCTD could induce colon cancer cell apoptosis through the αvβ6-ERK signaling pathway^4^; however, the mechanism by which the signal was transduced to the cell nucleus and the related downstream factors involved must be further investigated. We also found that the morphology of colon cancer cells changed after treatment with NCTD, and this change was similar to the change that occurred during the mesenchymal-epithelial transition (MET). MET is the reverse of the process of epithelial-mesenchymal transition (EMT), which is a crucial step in regulating the malignant behaviors of colon cancer cells.

In the present study, the human colon cancer cell lines HT-29 and WiDr were used to investigate the effects of NCTD on the cellular EMT process and the signaling pathways involved in this process.

## Materials and Methods

### Cell lines and culture conditions

The human colon cancer cell lines HT-29 and WiDr were obtained from the American Type Culture Collection (ATCC) and maintained as monolayers in standard Dulbecco’s modified Eagle’s medium (DMEM: 4.5 g/l glucose) (Sigma, USA) with 10% heat-inactivated fetal calf serum (FCS) (Sigma, USA), 20 mM HEPES, 100 IU/ml penicillin and 100 μg/ml streptomycin (Merck, Germany). The cells were incubated at 37 °C with 5% CO_2_ and saturated humidity. Cells in the exponential phase of growth (density of 2 × 10^5^ cells/ml) were exposed to various doses of NCTD for different time intervals. The negative control culture was left untreated. Changes in cellular morphology were observed with a phase-contrast microscope (Leica, Germany).

### Antibodies and reagents

The mouse-anti-human monoclonal antibody R6G9 (IgG_2a_), which was directed against the extracellular domain of human integrin subunit β6, was obtained from Chemicon International (Temecula, CA, USA). Peptide IK2 (synthesized previously) was used to block the direct binding of β6 and ERK. Monoclonal antibodies HECD-1, 5D5, and VI-10, which targeted E-cadherin, N-cadherin, and vimentin, respectively, were obtained from Abcam (Cambridge, MA, USA). The following were also obtained from Abcam (Cambridge, MA, USA): monoclonal antibodies EP1238Y and EPR16623, which targeted the crucial Notch signaling pathway factors Notch1 and Notch3, respectively; monoclonal antibodies 3A6 and E247, which targeted the crucial Wnt signaling pathway factors Wnt3a and β-catenin, respectively; monoclonal antibodies Y89 and EP2109Y, which targeted the crucial PI3K-AKT signaling pathway factors AKT1 and p-AKT1, respectively; and monoclonal antibodies EP567Y, EPR2856(N), EP568Y, EP823Y, and Y89, which targeted the crucial TGF-β classical signaling pathway factors Smad2, p-Smad2, Smad3, and p-Smad3. Mouse immunoglobulins IgG_2a_ and IgG_1_ were acquired from DAKO (Copenhagen, Denmark). Antibodies against ERK, p-ERK, JNK, p-JNK, p38, and p-p38 were purchased from Santa Cruz Biotechnology (Santa Cruz, CA, USA). Monoclonal antibodies EP1186Y and EP1254, which targeted the MMP-3 and MMP-9, respectively, were purchased from Abcam (Cambridge, MA, USA). Reagents for SDS–polyacrylamide gel electrophoresis (SDS–PAGE) and molecular weight markers were obtained from Bio-Rad Laboratories (Hercules, CA, USA). NCTD of analytical grade purity was obtained from Beijing Fourth Pharmaceutical Works (Beijing, China).

### RNA extraction and RT-PCR analysis

Total cellular RNA was extracted from untreated and NCTD-treated cells using Trizol reagent (Sigma), and cDNA was synthesized according to the manufacturer’s instructions (Promega, Madison, WI, USA). Equal amounts of cDNA were subjected to PCR analysis. The sequences of the primers were as follows: β6 integrin forward: 5’-AGGATAGTTCTGTTTCCTGC-3’ and β6 integrin reverse: 5’-ATCATAGGAATATTTGGAGG-3’, which generated a 141-bp amplicon; MMP-3 forward: 5’-CGGTTCCGCCTGTCTCAAG-3’ and MMP-3 reverse: 5’-CGCCAAAAGTGCCTGTCTT-3’, which generated a 206-bp amplicon; MMP-9 forward: 5’-ACCTCGAACTTTGACAGCGAC-3’ and MMP-9 reverse: 5’-GAGGAATGATCTAAGCCCAGC-3’, which generated a 133-bp amplicon; β-actin forward: 5’-GAGACCTTCAACACCCCAGCC-3’ and β-actin reverse: 5’-AATGTCACGCACGATTTCCC-3’, which generated a 264-bp amplicon and was used as an internal control. The amplification conditions were 33 cycles of 94 °C for 30 s, 51 °C for 30 s, and 72 °C for 30 s. The PCR products were electrophoresed on a 1.5% agarose gel and viewed by ethidium bromide staining. Data were analyzed with Alpha Imager software (Alpha Innotech Co., CA, USA), and the expression levels of αvβ6 were normalized to the expression levels of β-actin.

### MMP activity assay

MMP-3 and MMP-9 levels in the conditioned medium obtained from untreated and NCTD-treated cells were assayed using a commercially available kit, the Biotrak MMP activity assay system (Amersham, Aylesbury, UK). This assay measured total MMP levels (inactive pro-enzyme activated artificially plus endogenous active enzyme forms), and MMP secretion was calculated on a per-cell basis.

### Transwell assay for cell invasion

The invasive ability of colon cancer cells was analyzed in 24-well Boyden chambers with polycarbonate membranes (8-μm pore size) (Costar, Acton, USA). The membranes were pre-coated with 50 μl of Matrigel (BD Biosciences, San Diego, USA) to form matrix barriers. Cells were resuspended in 100 μl of serum-free medium at a concentration of 1 × 10^6^ cells/ml and dispensed into the upper chamber; the lower compartments were filled with 600 μl of medium with 10% FBS. After incubation, the cells remaining on the upper surface of the membrane were removed. Cells on the lower surface of the membrane were fixed and stained with crystal violet and counted under a light microscope at ×200 magnification.

### Western blotting

HT-29 and WiDr cells were treated with various doses of NCTD for 24 h. Both adherent and floating cells were collected and frozen at −80 °C. To detect the levels of potential signaling pathway proteins, the proteins were extracted from the cells. The protein concentration was measured using the BCA protein assay reagent, and equal amounts of protein (10 μg) were loaded onto a 12.5% SDS-PAGE gel and electrophoresed under non-reducing conditions. After electrophoresis, the proteins were transferred to nitrocellulose membranes. Equivalent protein loading in each lane was reconfirmed by staining the nitrocellulose membrane with Ponceau using the 36-kDa GAPDH band as a reference marker. The membranes were then probed with primary polyclonal antibodies against the crucial signaling pathway factors followed by peroxidase-conjugated secondary antibodies. Proteins on the western blots were visualized using the enhanced chemiluminescence detection system according to the manufacturer’s instructions. The optical density was analyzed with the Image J software.

### Transcription factor activation profiling array

We used the transcription factor activation profiling plate array (synthesized by Signosis, Sunnyvale, CA) to analyze the activity of different transcription factors in HT-29 and WiDr colon cancer cells treated with NCTD for 24 h according to the manufacturer’s instructions. First, nuclear proteins were extracted from HT-29 and WiDr cells using a Nuclear Extraction Kit (Signosis, Sunnyvale, CA). Then, 15 μg of nuclear protein extract was mixed with biotin-labeled probes based on the consensus sequences of transcription factor DNA binding sites. Thus, transcription factor/probe complexes were formed. Next, we separated the transcription factor-DNA complexes from free probes, transferred the complexes to a PCR tube, and denatured the eluted probes. After separation of the bound probes from the complexes, they were hybridized with sequences complementary to the probes in the hybridization plate. Finally, the captured DNA probes were detected with streptavidin-HRP, and the signal intensity was measured based on relative light units (RLUs) using a microplate luminometer.

### Immunofluorescence (IF)

Cells on cover slips were fixed, permeabilized and stained with tetramethylrhodamine (TRITC)-conjugated phalloidin (Sigma-Aldrich, St. Louis, USA) for 1 h. Then, 4′,6-diamidino-2-phenylindole (DAPI) (Beyotime) was used for nuclear staining. Cells were observed under a confocal laser microscope (Carl Zeiss, LSM780, Oberkochen, Germany). The “% decrease in F-actin” was calculated as follows: [(F-actin in untreated cells–F-actin in NCTD treated cells)/F-actin in untreated cells] ×100.

### Statistical analysis

The results were expressed as the means ± SD. Comparisons between the two groups were performed with Student’s *t* test. Comparisons of multiple samples and rates were carried out using single-factor analysis of variance. All statistical analyses were performed using SPSS for Windows version 16.0. *P* values <0.05 were considered statistically significant.

## Results

### NCTD treatment induced Morphological changes of colon cancer cells

**Figure 1:**
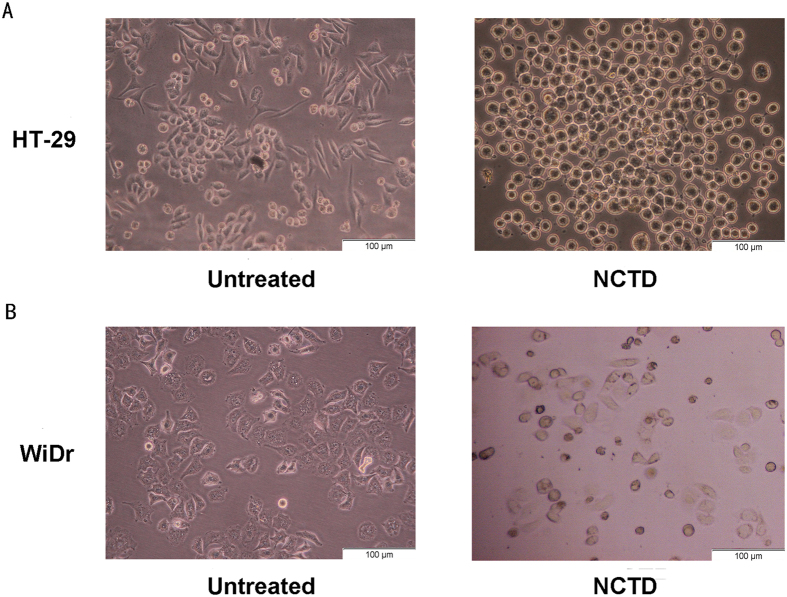
Morphological changes in HT-29 and WiDr colon cancer cells after NCTD treatment HT-29 and WiDr colon cancer cells were pretreated with 40 μmol/L NCTD in culture medium for 24 h. (**A**) HT-29 colon cancer cells had a fibroblast-like morphology before treatment with NCTD, but they became round quickly after treatment with NCTD. (**B**) The same phenomenon was also observed in WiDr cells after treatment with NCTD.

To investigate the effect of NCTD on cell morphology, we pre-tested a series of NCTD concentrations (from 0 to 60 μmol/L) at different times. The results of these preliminary assays showed that the morphological changes associated with MET were most obvious when the cells were treated with 40 μmol/L NCTD for 24 h. Therefore, we treated the HT-29 and WiDr colon cancer cells with 40 μmol/L NCTD for 24 h. As shown in Fig. 1, HT-29 and WiDr colon cancer cells had a spindle- or fibroblast-like morphology prior to treatment with NCTD, but most cells exhibited a typical round or oval shape after treatment with NCTD. This morphology change was similar to what had been shown to occur during MET, which was the reverse process of EMT.

### NCTD markedly suppressed the invasive capacity of colon cancer cells

**Figure 2:**
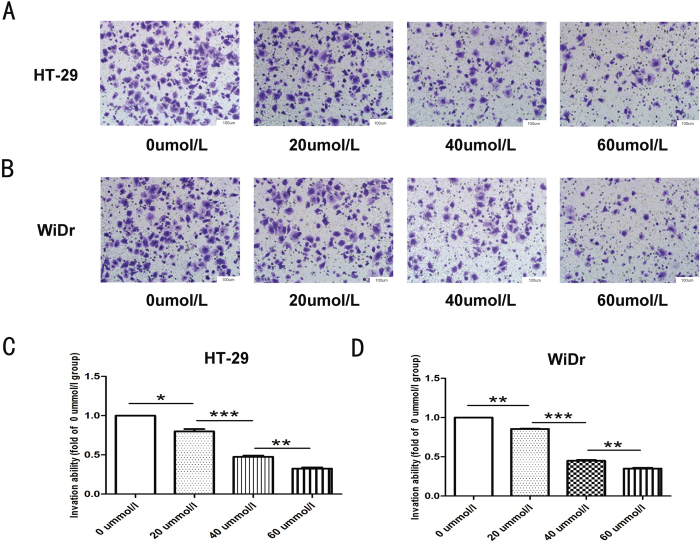
NCTD inhibited the invasiveness of colon cancer cells (**A**) NCTD can significantly reduce the invasion ability of HT-29 cells in a dose-dependent manner. (**B**) NCTD can significantly reduce the invasion ability of WiDr cells in a dose-dependent manner. (**C,D**) Statistical analysis confirmed that NCTD could inhibit the invasion ability of colon cancer cells. Results are shown as the mean ± SD of invaded cells in three independent experiments. *p < 0.05, **p < 0.01, ***p < 0.001.

To evaluate the effect of NCTD on colon cancer cell invasion, transwell assays were carried out. The results showed that compared with no treatment, NCTD treatment significantly reduced the number of HT-29 (Fig. 2A) and WiDr (Fig. 2B) colon cancer cells that invaded through the Matrigel-coated membrane in a dose-dependent manner (Fig. 2C,D).

### NCTD suppressed the expression of αvβ6, MMP-3, and MMP-9 and the polymerization of F-actin in colon cancers

**Figure 3:**
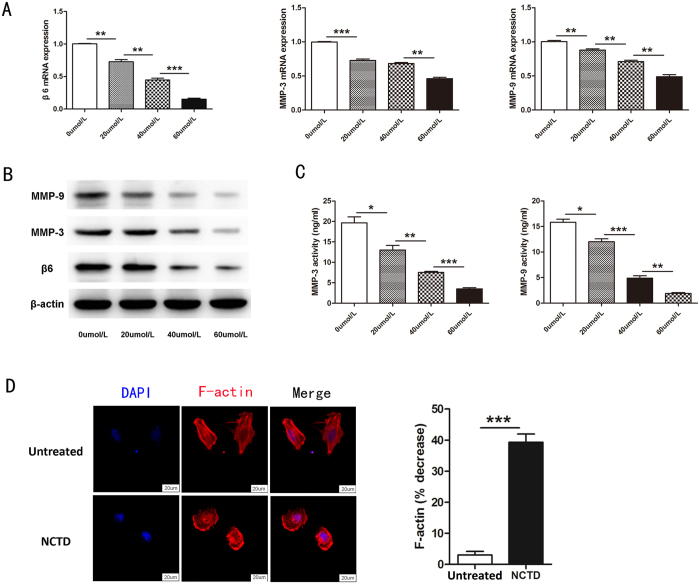
NCTD reduced the expression of αvβ6, MMP-3, and MMP-9 and the polymerization of F-actin. (**A**) RT-PCR analysis of αvβ6, MMP-3 and MMP-9. After colon cancer cells were treated with different doses of NCTD, the mRNA levels of αvβ6, MMP-3 and MMP-9 were decreased. Densitometry values are shown as the mean ± SD of three independent determinations. (**B**) Representative western blotting analysis of αvβ6 expression and secretion of MMP-3 and MMP-9 for 24 h after NCTD treatment. The expression of αvβ6, MMP-3 and MMP-9 was significantly decreased. (**C**) An MMP activity assay further showed that MMP-3 and MMP-9 activity was significantly and dose-dependently decreased in colon cancer cells treated with NCTD. (**D**) After treatment with NCTD, microfilaments in HT-29 cells were stained with tetramethylrhodamine (TRITC)-conjugated phalloidin and observed under a laser confocal microscope. The decrease in F-actin was analyzed compared with that in untreated cells. The results are shown as the mean ± SD of invaded cells in three independent experiments. *p < 0.05, **p < 0.01, ***p < 0.001.

To evaluate the mechanism by which NCTD inhibited invasion, we treated colon cancer cells with different doses of NCTD for 24 h, and we subsequently performed RT-PCR and western blotting to detect mRNA and protein levels, respectively. The RT-PCR results showed that NCTD significantly reduced the mRNA levels of αvβ6, MMP-3 and MMP-9. The inhibitory effect of NCTD was dose dependent (Fig. 3A). Moreover, western blotting showed that NCTD significantly reduced the expression of αvβ6, MMP-3 and MMP-9 at the protein level, also in a dose-dependent manner (Fig. 3B). To further investigate whether NCTD could interfere with the activity of MMP-3 and MMP-9, an MMP activity assay was performed. When colon cancer cells were treated with 20, 40 and 60 μmol/L NCTD for 24 h, the activity of MMP-3 and MMP-9 decreased substantially in a dose-dependent manner (Fig. 3C).

It is well known that cytoskeletal reorganization is essential in cell motility and tumor metastasis. Microfilaments (also referred to as actin filaments or F-actin) are helical polymers of globular actin subunits and are the thinnest fibers of the cytoskeleton. It is known that actin filaments provide mechanical support for the cell, determine the cell shape, enable cell movements, are associated with certain cell-cell junctions, and participate in cytoplasmic streaming and contraction of the cell during cytokinesis. Therefore, we investigated the organization of microfilaments in cells treated with NCTD. Immunofluorescence results (shown in Fig. 3D) revealed that NCTD could significantly induce the depolymerization of cellular microfilaments (F-actin), causing changes in cellular morphology and the redistribution of F-actin. However, there were no obvious changes in the untreated group. These results might account for the inhibitory effect of NCTD on colon cancer cell migration.

### The effect of NCTD on EMT-related proteins in colon cancer cells

**Figure 4:**
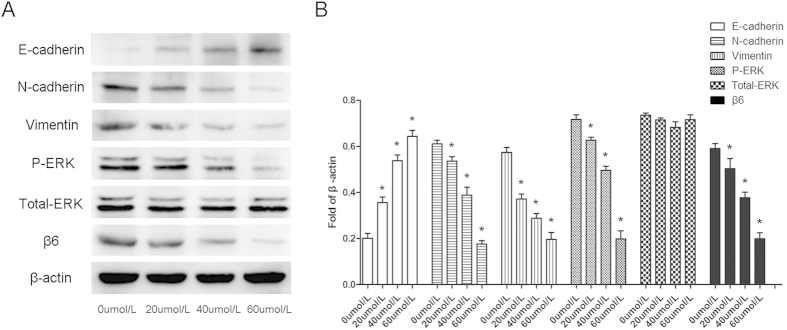
NCTD induced a MET process in colon cancer cells. (**A**) HT-29 colon cancer cells were pretreated with 20, 40 and 60 μmol/L NCTD for 24 h. Then, the expression of representative molecules of EMT was detected. The expression level of the epithelial marker E-cadherin increased, while the mesenchymal markers N-cadherin and vimentin decreased. Moreover, NCTD could suppress the expression of p-ERK in a dose-dependent manner as well. (**B**) The expression of representative molecules in the western blotting was expressed as a fold of β-actin. The results are shown as the mean ± SD of three independent experiments. *P < 0.05.

After treatment with NCTD, colon cancer cells underwent a MET-like process, and we detected some typical MET-related molecules. We found that the expression level of the epithelial marker E-cadherin increased, while the mesenchymal markers N-cadherin and vimentin showed decreased expression (Shown in Fig. 4). All changes were dose dependent, indicating that NCTD could induce the MET process in colon cancer cells.

Classical signaling pathways involved in the cellular EMT process include the TGF-β, MAPK, Notch, Wnt, and AKT pathways. After colon cancer cells were treated with 20, 40 and 60 μmol/L NCTD for 24 h, we detected the expression of proteins that were representative of the aforementioned classical signaling pathways. The results showed that only the protein level of p-ERK decreased, and no obvious variations in the other proteins were observed (Fig. 4 and Supplemental Fig. 1).

### NCTD induced the MET process in colon cancer cells via the αvβ6-ERK-Ets1 signaling pathway

**Figure 5:**
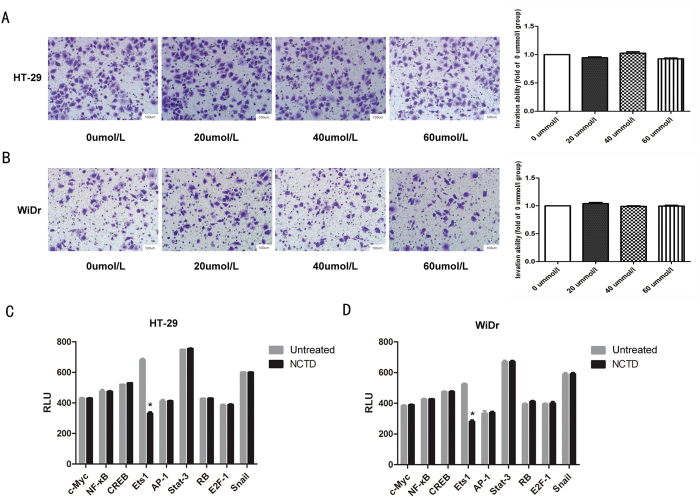
NCTD functioned through the αvβ6-ERK-Ets1 signaling pathway. (**A,B**) HT-29 (**A**) and WiDr (**B**) colon cancer cells were pretreated with peptide IK2, and the cells were treated with NCTD at different concentrations. A transwell assay was performed to detect the invasion capability. (**C,D**) HT-29 (C) and WiDr (D) colon cancer cells were pretreated with 40 mol/L NCTD in culture medium for 24 h. Then, we detected the downstream targets of ERK2 to observe the variation in transcription factor activity in HT-29 and WiDr cells. The results are shown as the mean ± SD of invaded cells in three independent experiments. *p < 0.05. Supplemental Fig. 1. Detecting the expression of EMT-related proteins involved in classical signaling pathways after treatment with NCTD HT-29 colon cancer cells were pretreated with 20, 40 and 60 μmol/L NCTD for 24 h. Then, the expression of mean EMT-related proteins involved in classical signaling pathways was detected. The results showed that there were no significant changes in these pathways.

We found that αvβ6 promoted malignancy of colon cancer cells by binding with ERK and subsequently activating the downstream signaling pathways^5^. To investigate the mechanism by which NCTD mediated MET in colon cancer cells, we treated the colon cancer cells with peptide IK2, which specifically blocks the direct binding of β6 and ERK. The cells were treated with different concentrations of NCTD, and a transwell assay was performed to detect their invasion capability. Interestingly, after treatment with IK2, NCTD had no effect on the invasion ability of colon cancer cells, indicating that NCTD suppressed the invasion ability of colon cancer cells by interfering with the direct connection between β6 and ERK (Fig. 5A,B). Next, we investigated the downstream factors involved in this process. HT-29 colon cancer cells were pretreated with 40 μmol/L NCTD for 24 h. Then, we examined downstream targets of ERK2 to observe variations in the activity of transcription factors (including c-Myc, NF-kappaB, CREB, Ets1, AP-1, Stat-3, RB, E2F-1, and Snail) in HT-29 and WiDr cells. The results showed that the activity of the transcription factor Ets1 was significantly inhibited in HT-29 cells treated with NCTD (*P* < 0.05), but it had no significant effect on the activity of other transcription factors (Fig. 5C). Similar results were found in WiDr cells (Fig. 5D).

## Discussion

EMT is the process by which the cell phenotype changes from epithelial to mesenchymal. It is widely accepted that the EMT process plays an important role in regulating malignant epithelial tumor cell invasion, and it is the key step in distant tumor metastasis. During the process of EMT, the expression of epithelial protein markers, such as E-cadherin, decreases, and the expression of mesenchymal protein markers, such as vimentin and N-cadherin, is up-regulated^6^. Researchers have previously confirmed that EMT is involved in the invasion and metastasis process in malignant epithelial tumor tissues^7^.

The process is regulated at the transcriptional and post-transcriptional levels, and it involves many signaling pathways^8^. Researchers have found that different Smads can mediate the EMT process via the classical TGF/Smad signaling pathway in colon cancer, hepatocarcinoma, and mammary adenocarcinoma^9^,^10^. In addition, TGF-β can also activate TGF-β-activated kinase 1 and subsequently activate RAS homolog gene family member A (RhoA) or other factors, hence mediating the EMT process via non-Smad signaling pathways^11^. Howard^12^ confirmed that Wnt could combine with the transmembrane receptor Frizzled, interfered with the degradation of β-catenin, and promoted the entry of β-catenin into the cell nucleus, which could activate Wnt target genes, including Ets, Jun, and Slug, thus promoting the EMT process. Yan^13^ speculated that the EMT process in liver cancer cells was induced by hypoxia and was regulated by the PI3K-AKT signal pathway. When hepatocellular carcinoma and pancreatic cancer cells undergo EMT^14^,^15^, the Notch receptor, which is activated by its ligand, splits into two parts. The intracellular portion of Notch is released and enters the cell nucleus, subsequently regulating the transcription of downstream target genes, including members of the basic helix-loop-helix (bHLH), hairy and enhancer of split (HES) and HES-related inhibitory protein families as well as the zinc finger transcription factors (e.g., Snail and Slug). Secker^16^ showed that the mitogen-activated protein kinase (MAPK) signaling pathway also regulated the EMT process. There are two major pathways participating in the MAPK regulation: Ras/Raf/MAPK and P38 Jun^17^,^18^.

Integrin is a transmembrane glycoprotein receptor consisting of a non-covalently bonded α and β subunit, and it belongs to the cell surface adhesion molecular family. αvβ6 is a special integrin subtype that is only expressed in epithelial cells, and its major ligand is fibronectin (FN). In normal epithelial cells, the expression of αvβ6 is rare and can hardly be detected^19^, but it increases substantially in response to injury and/or inflammation and in epithelial tumors (gastric carcinoma, colon cancer, and others)^20^,^21^,^22^. The *de novo* expression of integrin αvβ6 has been shown to modulate several behaviors of colon carcinoma cells, including cell adhesion and spreading on fibronectin, proliferation in collagen gels, tumor growth, cell invasion and metastasis, and cell apoptosis, which we demonstrated throughout our 20-year investigation of integrin αvβ6^23^,^24^,^25^,^26^.

Importantly, we have previously shown a direct physical linkage between ERK2 and the cytoplasmic domain of β6, ^746^EAE*RSKAKWQTGTNPLYR*G^764^ (the ERK2 binding sequence is italicized)^27^. Through the direct linkage between αvβ6 and ERK2, integrin αvβ6 transmits out-in signals accompanied by an increase in the phosphorylation level of ERK2, influencing many of the malignant activities of cancer cells^28^. Effective targets of phosphorylated ERK2 are found to be localized in the nucleus.

It is known that the direct linkage between αvβ6 and ERK2 can increase the phosphorylation level of ERK2 in colorectal cancer cells, and activated ERK 1/2 can increase Ets-1 transcriptional activity by forming a signaling complex with Ets-1, which could enhance binding to the promoter region and induce transcriptional activation of genes involved in malignancy^29^,^30^. In this study, we found that after colon cancer cells were treated with NCTD for 24 h, the protein level of p-ERK decreased, accompanied by an increase in the epithelial marker E-cadherin and a decrease in the mesenchymal markers N-cadherin and vimentin. However, other key proteins in the EMT process, such as Notch1 and Notch3 in the Notch signaling pathway, Wnt3a and β-catenin in the WNT signaling pathway, Akt in the PI3K signaling pathway, and Smad2 and Smad3 in the TGF-β signaling pathway, showed no obvious variation. We treated the colon cancer cells with peptide IK2, specifically blocking the direct binding of β6 and ERK. Interestingly, after treatment with IK2, NCTD had no effect on the invasion ability of these cells, indicating that NCTD inhibited the invasion ability of colon cancer cells through the direct connection between β6 and ERK. Furthermore, the transcription factor p-ERK was investigated via an activation profiling array, which showed that only the activity of the transcription factor Ets1 was significantly inhibited in colon cancer cells treated with NCTD. Thus, we demonstrated that NCTD induced the MET process of colon cancer cells via the αvβ6-ERK-Ets1 signaling pathway. NCTD suppressed the migration and invasion ability of colon cancer cells in a dose-dependent manner.

The Ets-1 transcription factor contains an approximately 85-amino-acid DNA-binding domain, which binds to special purine-rich DNA sequences with a core motif of GGAA/T. Ets-1 transcriptionally regulates a number of viral and cellular genes, which are critical for several biological processes, including cellular proliferation, differentiation, development, transformation, and angiogenesis^31^,^32^.

In our previous study of NCTD, we preliminarily demonstrated that NCTD, as an anti-cancer traditional Chinese medicine, could decrease αvβ6 expression and inhibit ERK phosphorylation in HT-29 cells^4^. In this study, we found that NCTD could inhibit the EMT process of colon cancer cells, and it could cause colon cancer cells to switch from a spindle-shaped morphology to a round morphology. NCTD suppressed the migration and invasion ability of colon cancer cells in a dose-dependent manner. Meanwhile, the western blotting analysis and transcription factor activation profiling array confirmed that ERK and Ets1 participated in the signaling pathway. In light of these findings and our previous results, we propose that NCTD can inhibit the EMT process in colon cancer cells by interrupting the αvβ6-ERK-Ets1 signaling pathway. However, it is possible that another signaling pathway may participate in the NCTD-dependent inhibition of the EMT process because some cancer cells do not express αvβ6; this possibility needs to be investigated further. In addition, this study suggests that targeting any key protein in the αvβ6-ERK-Ets1 signaling pathway, combined with NCTD treatment, may serve as a potential therapeutic strategy for epithelial cell cancers expressing integrin αvβ6.

## Additional Information

**How to cite this article**: Peng, C. *et al.* Norcantharidin Suppresses Colon Cancer Cell Epithelial-Mesenchymal Transition by Inhibiting the avß6-ERK-Ets1 Signaling Pathway. *Sci. Rep.* **6**, 20500; doi: 10.1038/srep20500 (2016).

## Supplementary Material

Supplementary Information
