# Supplementary material for: Norcantharidin Suppresses Colon Cancer Cell Epithelial-Mesenchymal Transition by Inhibiting the αvβ6-ERK-Ets1 Signaling Pathway
Source: Sci Rep. 2016 Feb 5;6:20500. doi: 10.1038/srep20500 (PMC4742802; doi:10.1038/srep20500)

## Title Page

## Title: Norcantharidin Suppresses Colon Cancer Cell Epithelial-Mesenchymal Transition by Inhibiting the αvβ6-ERK-Ets1 Signaling Pathway

**Author:** Cheng Peng^a^, Zequn Li^a^, Zhengchuan Niu^a^, Wei Niu^a^, Zongquan Xu^b^, Huijie Gao^a^, Weibo Niu^a^, JiaYong Wang^a^, Zhaobin He^a^, Chao Gao^a^, Pengfei Lin^a^, Michael Agrez^c^, Zongli Zhang^a*^ and Jun Niu^a*^

**Authors’ affiliations:**

**^a^***Department of General Surgery, QiLu Hospital, Shandong University, Jinan, Shandong, China*

**^b^***Department of Hepatic Oncology, Jiangxi Provincial Cancer Hospital, Nanchang, Jiangxi, China*

**^c^***Newcastle Bowel Cancer Research Collaborative, The University of Newcastle, Callaghan, New South Wales, Australia*

***Corresponding author:**

Jun Niu and Zongli Zhang are co-corresponding authors.

Postal address: *Department of General Surgery, QiLu Hospital, Shandong University, Jinan 250012, Wenhuaxi Road 44#, Shandong, China.*

Tel: +86 531 82166651; Fax: +86 531 82169243.

E-mail addresses: Jun Niu : [niusdu120@163.com](mailto:niusdu120@163.com);

Zongli Zhang: [752215423@qq.com](mailto:752215423@qq.com)


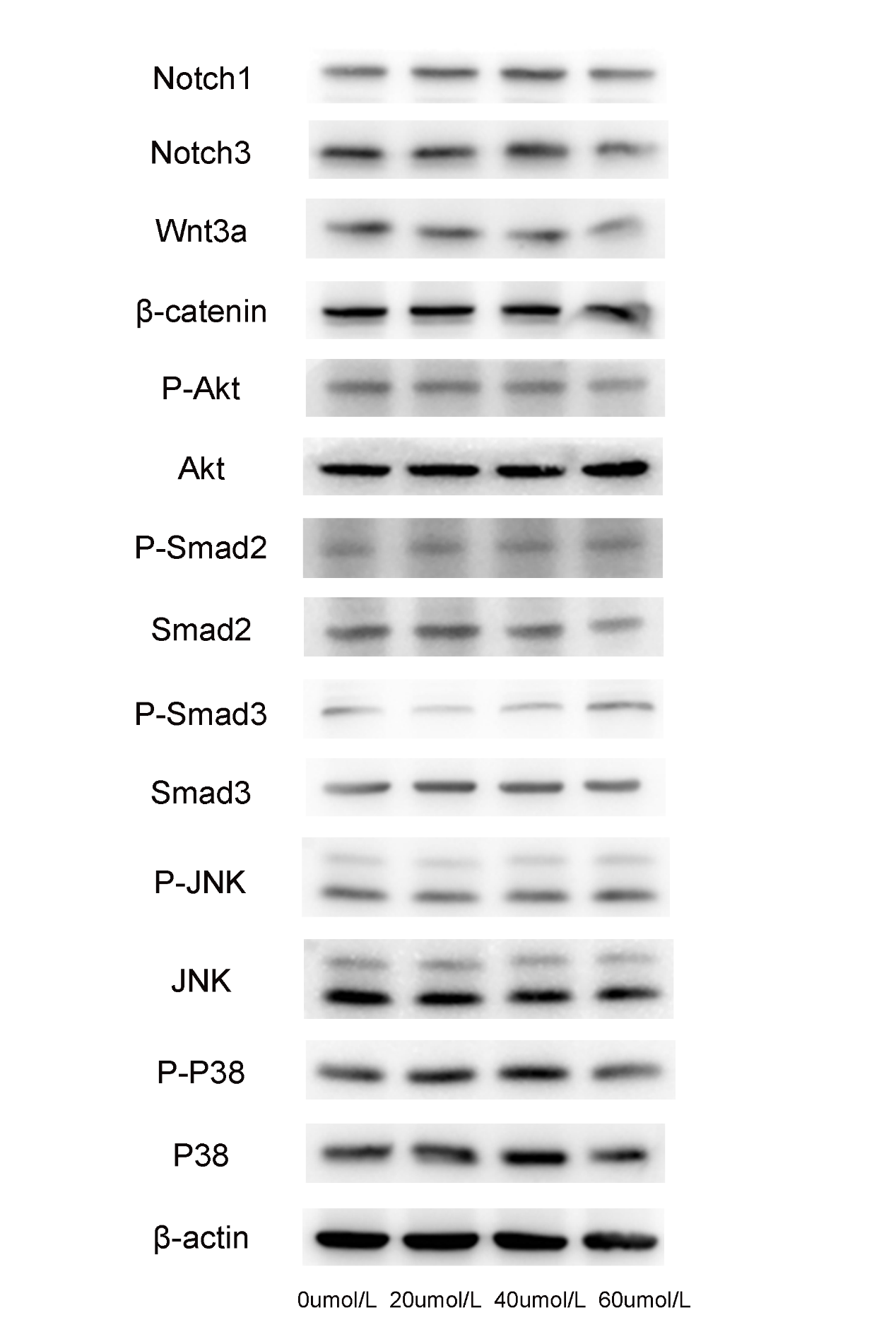
Supplemental figure 1

Supplemental Figure 1. **Detecting the expression of EMT-related proteins involved in** **classical signaling pathways after treatment with NCTD**

*HT-29 colon cancer cells were pretreated with 20, 40 and 60 μmol/L NCTD for 24 h. Then, the expression of mean EMT-related proteins involved in classical signaling pathways was detected. The results showed that there were no significant changes in these pathways.*

Following figures are the original data of western blotting results shown in figure 3, figure 4 and supplemental Figure 1:


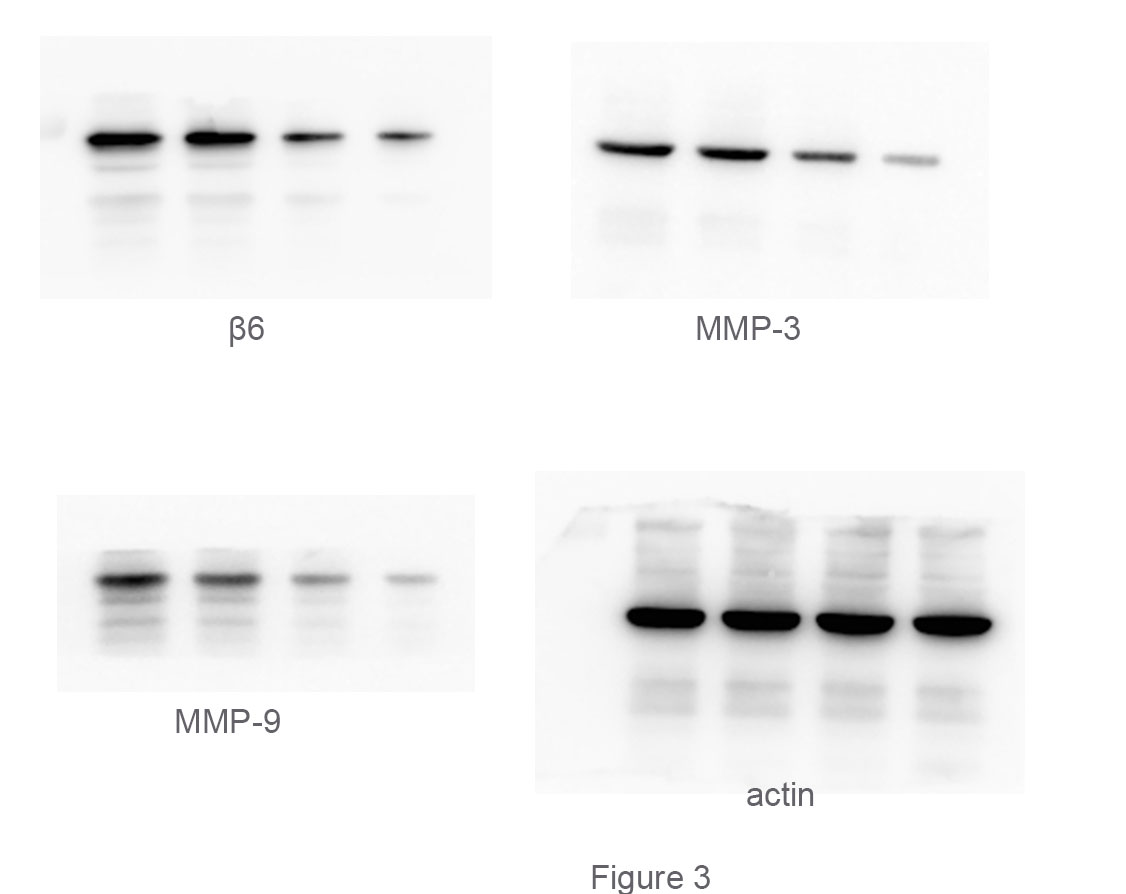


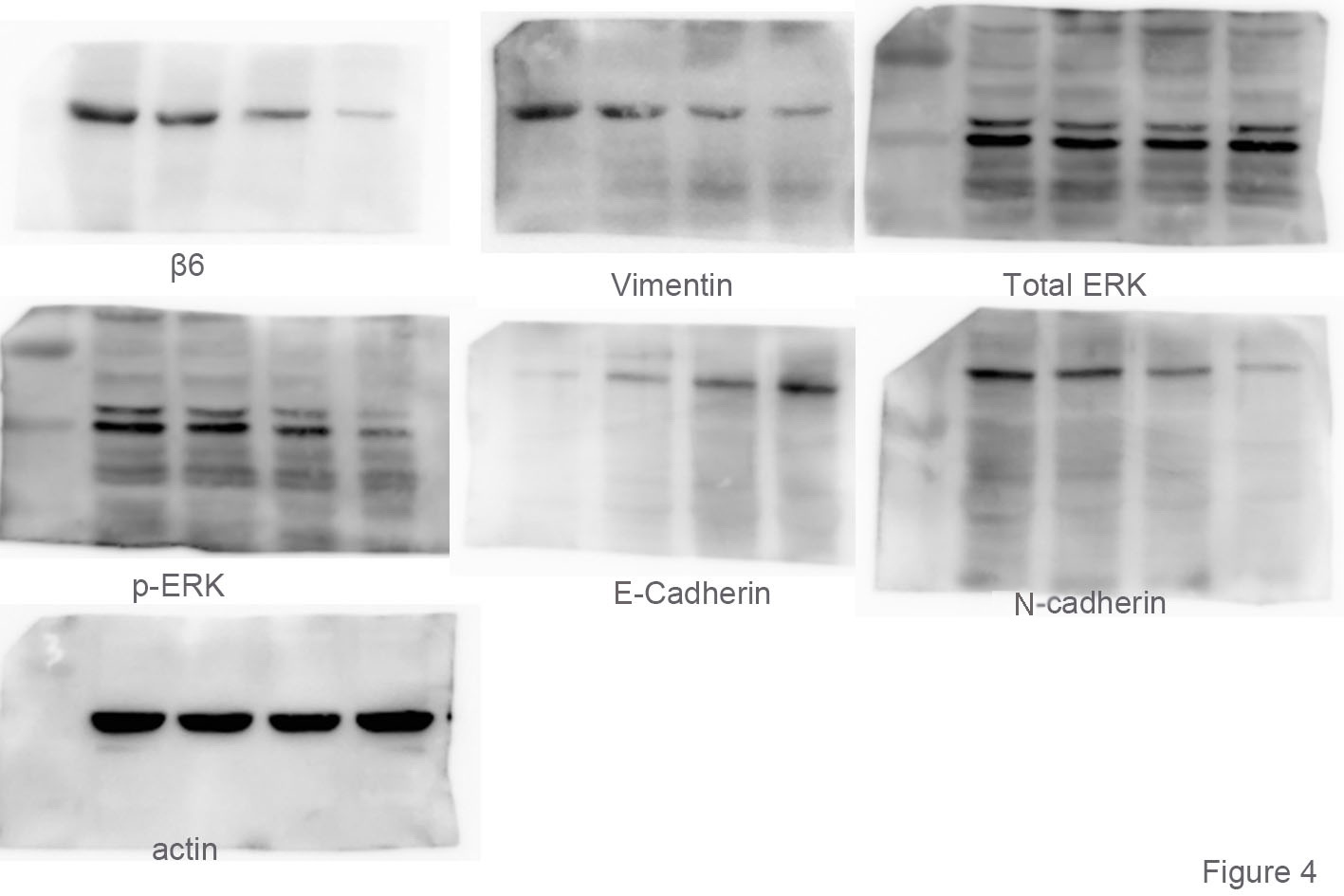


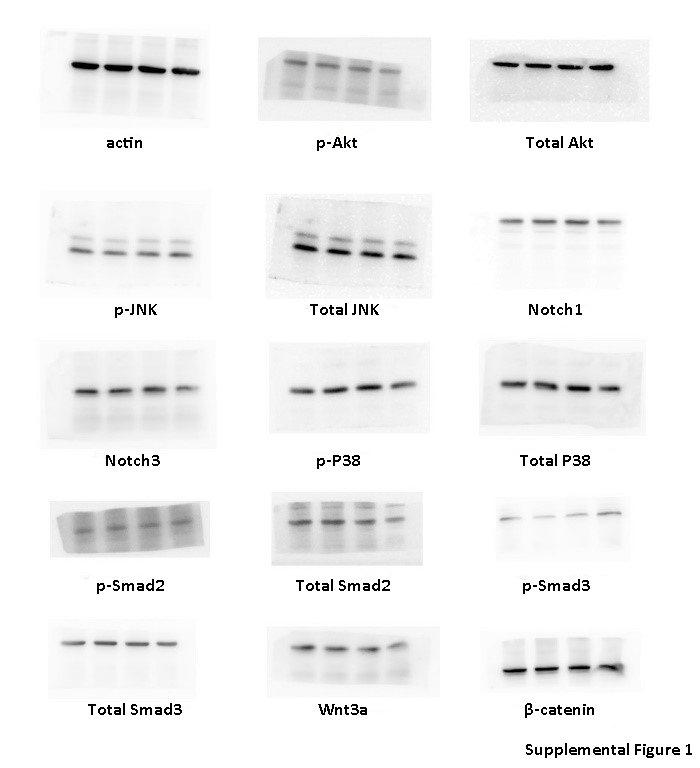

Supplement: Supplementary Information [file srep20500-s1.doc]
